# *UMOD* and the architecture of kidney disease

**DOI:** 10.1007/s00424-022-02733-4

**Published:** 2022-07-26

**Authors:** Olivier Devuyst, Murielle Bochud, Eric Olinger

**Affiliations:** 1 Institute of Physiology, University of Zurich, 8057 Zurich, Switzerland; 2 Center for Primary Care and Public Health (Unisanté), University of Lausanne, 1010 Lausanne, Switzerland; 3 Translational and Clinical Research Institute, Newcastle University, Newcastle upon Tyne, NE1 3BZ UK

## Abstract

The identification of genetic factors associated with the risk, onset, and progression of kidney disease has the potential to provide mechanistic insights and therapeutic perspectives. In less than two decades, technological advances yielded a trove of information on the genetic architecture of chronic kidney disease. The spectrum of genetic influence ranges from (ultra)rare variants with large effect size, involved in Mendelian diseases, to common variants, often non-coding and with small effect size, which contribute to polygenic diseases. Here, we review the paradigm of *UMOD*, the gene coding for uromodulin, to illustrate how a kidney-specific protein of major physiological importance is involved in a spectrum of kidney disorders. This new field of investigation illustrates the importance of genetic variation in the pathogenesis and prognosis of disease, with therapeutic implications.

With an estimated global prevalence above 10%, a high individual and societal burden, and limited therapeutic options, chronic kidney disease (CKD) is a major public health issue. Even in its early stages, CKD is associated with multiple complications and adverse outcomes, and it is a major risk factor for accelerated cardiovascular disease and aging [15]. For decades, the lack of mechanistic understanding of the multiple functions ensured by the kidney slowed the development of pharmacologic interventions targeting CKD and its associated complications [10, 32].

The familial clustering of kidney disorders, the high heritability of kidney function parameters, and the variable susceptibility of inbred animal strains to kidney damage drive strong efforts to decipher the genetic architecture of kidney diseases [5, 15, 37, 60]. The first genetic breakthroughs in nephrology were the mapping of autosomal dominant polycystic kidney disease (ADPKD) in 1985 [51] and the identification of a mutation in *COL4A5* causing Alport syndrome in 1990 [1]. Identification of genes involved in classic glomerular and tubular diseases quickly followed, including steroid-resistant nephrotic syndrome, nephropathic cystinosis, Dent disease, Bartter and Gitelman syndromes, nephrogenic diabetes insipidus, and ADPKD [10]. In less than two decades, the advent of new technologies including next-generation sequencing (NGS) and genome-wide association studies (GWAS) led to the identification of hundreds of genes causing inherited kidney disorders or associated with kidney function metrics and risk of CKD. In turn, these insights improved our understanding of processes operating in the normal and diseased nephron segments, thus providing novel therapeutic targets [10, 60, 67]. These discoveries were made possible thanks to collaborative studies gathering families with rare disorders and large, multi-ethnic cohorts for discovery of rare and common genetic variants, respectively. In this brief review, we will use the paradigm of genetic variation in *UMOD*, the gene coding for uromodulin—the most abundant protein in the kidney and in the normal urine—to illustrate how genetic approaches have the potential to yield insights into the architecture of CKD. These studies involved several Swiss cohorts, ranging from normal population to rare diseases, and technological developments supported by the Swiss National Center of Competence Kidney.CH.

## Spectrum of allele frequency and effect size in disease

**Fig. 1:**
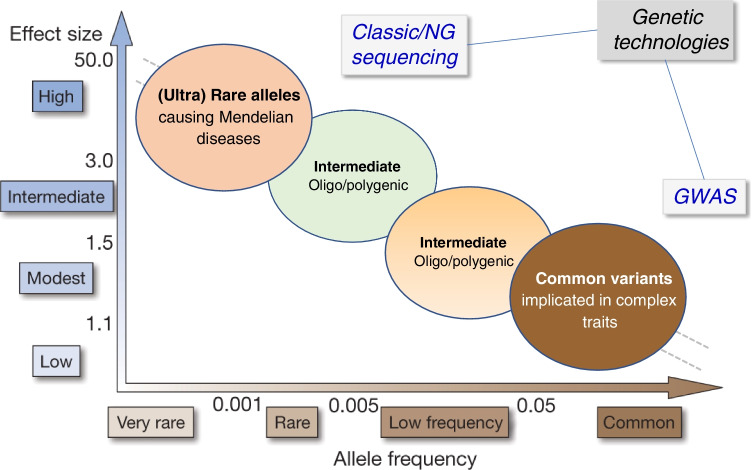
Genetic architecture of disease: spectrum of allele frequency and effect size. Variants in important genes may be involved in a continuum between rare and complex diseases, according to the risk allele frequency in the population (x-axis) and the strength of the effect size (odds ratio, y-axis). (Ultra)-rare alleles can be identified by next-generation sequencing (NGS), whereas common variants can be identified using genome-wide association studies (GWAS). Intermediate-size effect variants are predicted to complete the dichotomy, manifesting as a non-fully penetrant Mendelian disease or an oligo/polygenic model modifying disease expressivity. Figure adapted from Manolio et al. [34]

Once the genetic influence on a specific aspect of kidney function has been established, specific investigations can decipher the genetic architecture of disorders related to that trait. Three criteria are important when considering disease-associated allelic variants: (i) the frequency of the variant in the population; (ii) the effect size of the variant on the phenotype; and (iii) the number of genetic variants acting on the phenotype [52]. These criteria are classically represented on a X–Y plot, with the prevalence of the genetic variant on the x-axis and the magnitude of its effect size on the y-axis (Fig. 1). This relationship visualizes the spectrum of allelic variation, going from (ultra-) rare variants (minor allele frequency < 1°/_00_) with large-effect size, involved in Mendelian disorders, to common variants (frequency > 5%), with small-effect size involved in common, polygenic disorders [34]. These genetic variants can be identified using new technologies including NGS and GWAS, respectively. Intermediate-effect variants, not captured by GWAS but amenable to sequencing approaches, are predicted to be part of the continuum (Fig. 1). Both population-based studies and rare disease cohorts are essential for such gene discovery.

## SKIPOGH and CoLaus cohorts: heritability and GWAS studies

**Fig. 2:**
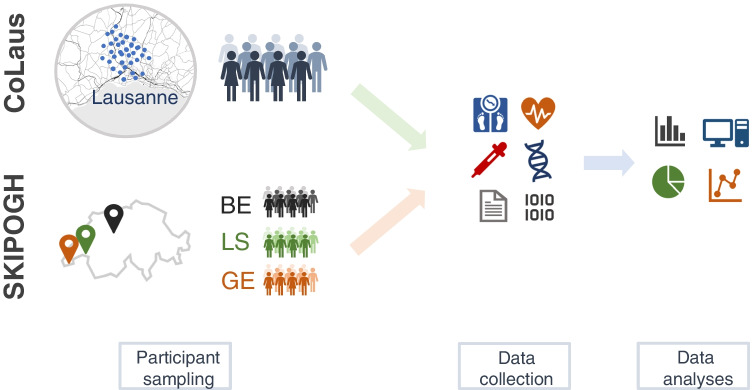
Infographics summarizing the design of the CoLaus and SKIPOGH population-based studies

Two population-based Swiss cohorts provided invaluable support to studies investigating heritability of kidney function paramaters and the role of *UMOD* variations in CKD (Fig. 2).

The CoLaus study (“Cohorte Lausannoise”) is a longitudinal, population-based cohort initiated in 2003 in the city of Lausanne. The CoLaus participants were randomly selected from the complete list of Lausanne inhabitants in 2003 [16]. Inclusion criteria were (i) written informed consent; (ii) age 35–75 years; (iii) willingness to take part in the examination and donate a blood sample; and (iv) Caucasian origin. By the end of the recruitment in 2006, the baseline CoLaus dataset included 6538 participants (participation rate: 34%). Participants were first seen in 2003 to 2006, then followed-up from 2009 to 2012, 2014 to 2017, and 2018 to 2021. At all four visits, participants attended a morning medical examination at the Lausanne University Hospital after an overnight fast, which comprised a blood sampling, assessment of anthropometric features, and completion of a detailed health questionnaire inquiring about lifestyle factors, socioeconomic and marital status, personal and family history of disease, as well as medication intake [39]. The CoLaus project includes a series of “sub-studies” aimed at exploring specific health-related outcomes and environmental exposures (https://www.colaus-psycolaus.ch/).

The Swiss Kidney Project on Genes in Hypertension (SKIPOGH) is a family- and population-based cohort investigating the genetic and environmental determinants of renal function and blood pressure. SKIPOGH is a multicenter, longitudinal study with participants recruited in the city of Lausanne, and the cantons of Geneva and Bern [46, 49]. At inception, eligible subjects were randomly selected from the population-based CoLaus study in Lausanne and from the population-based Bus Santé study in Geneva [26]. In Bern, subjects were randomly selected using the cantonal phone directory. Inclusion criteria were (i) written informed consent; (ii) minimum 18 years of age; (iii) European descent; and (iv) at least one, and ideally three, first degree family members willing to participate to the study. Participation rate was 20% in Lausanne, 22% in Geneva, and 21% in Bern. The first SKIPOGH wave took place between 2009 and 2013 (baseline: SKIPOGH 1), whereas the second wave began in 2012 and ended in 2016 (follow-up: SKIPOGH 2). SKIPOGH 1 included 1128 participants coming from 273 nuclear families. At both waves, study participants attended a morning medical visit after an overnight fast, which included blood sampling, kidney imaging, cardiac evaluation, and biobanking. Participants completed a detailed questionnaire about their medical history, medication intake, lifestyle factors, and socioeconomic characteristics (http://www.skipogh.ch/index.php/Welcome_to_the_SKIPOGH_study!).

Estimating the relative contribution of genetic and environmental influences on kidney function parameters and serum and urinary electrolytes levels is a prerequisite for studies aiming at identifying genes involved in kidney function. Thanks to the application of a standardized protocol across several centers, the heritability (i.e., the proportion of variance of a given trait explained by genetic effects) of estimated glomerular filtration rate (eGFR) as well as of serum and urine concentrations, renal clearances, and fractional excretions of major electrolytes could be analyzed in the family-based SKIPOGH cohort [37]. High heritability estimates were observed for GFR estimated using the CKD-EPI Eq. (46 ± 6%), serum creatinine (49 ± 6%), CKD-EPI cystatin C (58 ± 5%), and serum urea (49 ± 6%). The heritability of serum concentrations was highest for calcium, 37% and lowest for sodium, 13%. All probability values were significant. These results provided a basis for further investigations of the genetic basis of kidney function and electrolyte homeostasis in the general population.

In addition to the heritability studies, data from participants of the SKIPOGH and/or CoLaus cohorts were included in GWAS which identified new loci for kidney function and CKD [70], uromodulin production and excretion [29, 40], albuminuria [58, 59], serum urate levels [61], magnesium and calcium homeostasis [6, 7], and osmoregulation [3].

## *UMOD* variants in GWAS for kidney function and CKD

Genome-wide association studies are a useful, unbiased tool to uncover genomic regions (“loci”) associated with kidney function parameters and risk of CKD. Typically, GWAS require large sample sizes, yielding genome-wide significant (*P* value < 5 × 10^−8^) variants that are common and associated with a relatively small increase in disease risk [69]. The largest GWAS in the field of nephrology addressed traits like the eGFR based on serum creatinine (eGFRcrea) [13, 69, 70]. As discussed above, there is a strong genetic predisposition to CKD, as the heritability of the eGFR is close to 50% [37]. The largest and most recent GWAS for eGFR identified > 250 genetic loci and explained nearly 20% of that heritability [70]. Among those, variants in the *UMOD* locus display the largest effect size on the eGFR and CKD [70]. This effect, thought to be linked to the genetically driven expression of uromodulin (see below), is consistent across different ethnic groups and is also observed for longitudinal traits including rapid decline of eGFR [23].

The *UMOD* locus includes single-nucleotide polymorphisms (SNPs; e.g., rs12917707 and rs4293393) that are in complete linkage disequilibrium (LD) in a large block encompassing the gene promoter. For these SNPs, the “risk” allele is the common allele, with a frequency ranging between 0.765 and 0.955 [65]. Critically, the *UMOD* promoter variants are associated with the expression of uromodulin in the kidney and its levels in the urine and blood. In fact, homozygous carriers of the *UMOD* risk allele have twofold higher levels of uromodulin in the urine, compared with homozygous carriers of the (minor) protective allele [29, 40, 64]. Most recent GWAS for eGFR identified a strong, independent signal within *PDILT*, the gene flanking *UMOD* on chromosome 16 [70]. Of interest, the *PDILT* intronic variant rs77924615 is associated with uromodulin urinary levels, but not with the expression of *PDILT*, suggesting that it regulates uromodulin expression [70]. This hypothesis is supported by the independent association of rs77924615 with urinary uromodulin levels in two meta-GWAS [29, 40]. The possible links between uromodulin expression, which has been experimentally validated for the *UMOD-PDILT* variants, and other transcripts correlated with variants in the same locus have not been explored [31].

The *UMOD* locus has also been associated with hypertension and incident cardiovascular disease [44], kidney stones [25], and gout/uric acid [25, 61], strengthening its importance for a set of common diseases. In these GWAS, the *UMOD* alleles associated with higher uromodulin expression/levels are associated with increased risk of CKD, hypertension, and hyperuricemia, whereas they are protective for kidney stones [11, 53].

## Biological relevance of the *UMOD* locus for CKD

**Fig. 3:**
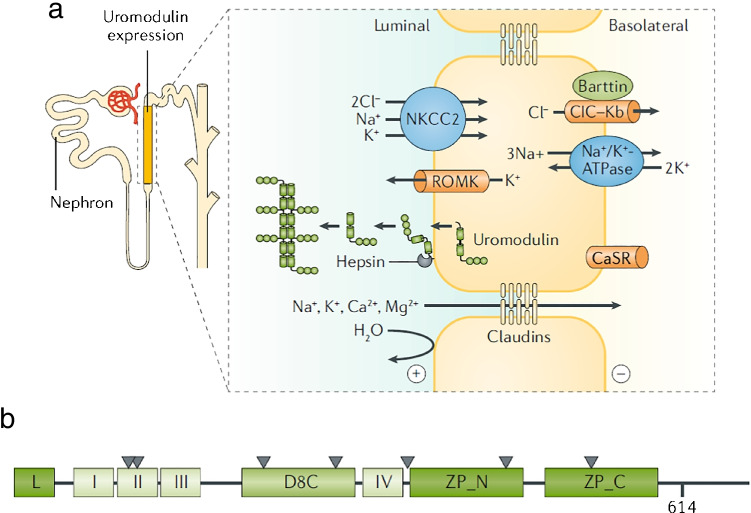
Site of production and structure of uromodulin. Uromodulin is mainly produced by the cells that line the thick ascending limb (TAL), a segment involved in the reabsorption of NaCl and divalent cations, while being not permeable to water. Uromodulin is a glycosylphosphatidylinositol (GPI)-anchored protein which traffics to the apical membrane of the cells, where it is cleaved by the serine protease hepsin and released in the urine where it forms large polymers. These polymers form the matrix of the urinary casts. The predicted structure of uromodulin contains a leader peptide (L); four EGF-like domains (I to IV); a cysteine-rich D8C domain; a bipartite C‑terminal Zona Pellucida domain (ZP_N and ZP_C) connected by a linker; and a GPI-anchoring site at position 614. The seven N‑glycosylation sites are indicated by triangles. Figure adapted from Devuyst et al. [11, 12]

The relevance of the *UMOD* GWAS locus in relation to kidney function is immediate, as *UMOD* is a kidney-specific gene coding for uromodulin, the most abundant protein expressed in the kidney and excreted in the normal urine [11, 13, 53]. Uromodulin is mainly produced by the thick ascending limb (TAL) of the loop of Henle and, to a lesser extent (about 10%) in the initial segment of the distal convoluted tubule (DCT) [63]. Uromodulin is a glycosylphosphatidylinositol (GPI)-anchored protein belonging to the family of zona pellucida (ZP) domain proteins (Fig. 3). The protein is heavily glycosylated (30% of the Mw) and it contains 48 conserved cysteine residues involved in 24 intramolecular disulfide bonds necessary for correct folding. Uromodulin contains four epidermal growth factor (EGF)-like domains, a cysteine-rich domain (D8C), and a bipartite ZP domain allowing protein polymerization. In TAL cells, the apical membrane-bond monomeric uromodulin is cleaved by the serine protease hepsin at a conserved cleavage site located near the C-terminus of the protein [4, 42]. The cleavage allows the polymerization of extracellular uromodulin into filaments forming a matrix-like structure in the urine, the main constituent of urinary casts [56]. Recently, the three-dimensional (3D) structure of native urinary uromodulin polymers was obtained by cryo-electron tomography, showing a zigzag-shaped backbone formed by polymerized ZP domains and protruding arms composed of the EGF and the D8C domains [68]. *N*-glycosylation mapping, biophysical assays, and imaging revealed that uromodulin binds the *Escherichia coli* type 1 pilus adhesin and that uromodulin filaments associate with uropathogens and mediate bacterial aggregation, potentially preventing adhesion and promoting clearance of the pathogens [68]. Recent studies also shed light on the regulation of uromodulin excretion in the urine, including a role played by the calcium-sensing receptor [62] and the type 1 keratin KRT40 [29], and the activity of transport processes operating in the TAL [50, 54, 62].

If the vast majority of uromodulin is apically targeted to form organized polymers in the urine, it should be noted that uromodulin is also detected in monomeric form in the serum, at levels that are at least 100-fold lower than in the urine [11, 53]. The mechanisms regulating the basolateral trafficking of uromodulin and its release into the interstitium and further into the circulation remain essentially unknown. A recent meta-GWAS of circulating uromodulin using different detection methods revealed that a missense variant (p.Cys466Arg) in the uromodulin-glycosylating enzyme B4GALNT2 was a loss-of-function allele leading to higher serum uromodulin levels, while other loci influence the glycosylation of circulating uromodulin [33]. The current view is that interstitial uromodulin may cross talk with proximal tubule cells, contributing to regulate innate immunity, whereas circulating uromodulin may act as an anti-oxidant [53].

Studies in uromodulin knock-out (*Umod*^−/−^) mice have provided key insights into the roles of uromodulin and its relevance for various processes operating in the kidney. A detailed description of these roles is provided in recent reviews [11, 53]. In brief, uromodulin polymers in the urine protect against kidney stone formation and urinary tract infections (UTIs); uromodulin regulates sodium transport systems in the TAL and DCT, thus regulating blood pressure and urinary concentrating ability; it stabilizes TRPV5, TRPV6, and TRPM6 channels, thus increasing their activity in the DCT. The small amount of uromodulin released from the basolateral side of the TAL cells into the interstitium may regulate processes in neighboring proximal tubule cells and contribute to innate response, e.g., by regulating the abundance and activity of resident mononuclear phagocytes, or enter the circulation and act as an antioxidant molecule [53]. All these processes are relevant when considering the onset of CKD, tubulointerstitial injury, and the complications arising from kidney failure.

The association of uromodulin expression levels with kidney damage was substantiated in a transgenic mouse model overexpressing wild-type uromodulin, mimicking the situation observed in carriers of the risk *UMOD* haplotype [65]. The transgenic *Umod* mice displayed a dose-dependent increase in systolic blood pressure, with salt-sensitive hypertension. Although kidney function remained normal, aging kidneys from the *Umod* transgenic mice showed focal lesions (e.g., tubular casts, cysts) and increased expression of kidney damage markers (e.g., lipocalin‑2, Kim‑1) and chemokines, that were not detected in the kidneys of control littermates. Similar focal lesions were evidenced in kidney biopsies from individuals aged > 65 years homozygous for the risk *UMOD* haplotype, compared to individuals homozygous for protective variants [65]. Together, these results suggest that, in subjects carrying the *UMOD* risk alleles, genetically driven higher production of uromodulin becomes deleterious over time, promoting the onset of CKD. The deleterious effect of higher uromodulin production in the kidney could be due to increased metabolic demand, chronic activation of salt reabsorption systems, or effects in the lumen of the distal tubule or in the circulation, explaining why these loci are associated with a strong age-effect [11, 13]. Of note, the *UMOD* risk variants associated with higher uromodulin levels are also associated with activated NKCC2, and thus higher response to furosemide [65]. A clinical trial testing the potential influence of these *UMOD* variants on the response to loop diuretics in individuals with hypertension is under way (ClinicalTrials.gov Identifier: NCT03354897).

## GWAS for urinary levels of uromodulin

The availability of reliable assays and protocols to measure uromodulin levels allowed to analyze the genetic factors associated with its excretion [71]. A first meta-GWAS approach performed on 10,884 individuals of European descent from six cohorts identified common variants within the promoter of *UMOD* as the single genome-wide significant locus associated with the levels of uromodulin in urine [40]. The top *UMOD* promoter variant, rs12917707, was associated in a dose-dependent fashion with the urinary levels of uromodulin, and in strong LD with the CKD variants [40].

A meta-analysis designed to increase the power to detect novel loci was recently conducted in 29,315 individuals of European ancestry from 13 cohorts including CoLaus [29]. Two genome-wide significant signals were identified for the urinary levels of uromodulin: a novel locus over the *KRT40* gene coding for keratin-40 (KRT40), a type 1 keratin expressed in the kidney, and the *UMOD-PDILT* locus, with two independent sets of single nucleotide polymorphisms spread over *UMOD* and the adjacent *PDILT*. In follow-up experiments, KRT40 was shown to colocalize with uromodulin in TAL cells, while knock-down of KRT40 expression in primary mTAL cells affected uromodulin processing and excretion, providing a biological counterpart for the GWAS association [29]. Keratins are intermediate filaments that form the cytoskeleton in epithelial cells. As cytoskeletal proteins, keratins are involved in maintaining the physical integrity, mechanical stability, and intracellular organization within cells—for example trafficking of proteins to the plasma membrane [8, 28]. That altered expression of KRT40 affects uromodulin (and also ROMK) processing in TAL cells suggest a role of specific cytokeratins on the sorting of proteins in kidney tubular cells [29].

## Uromodulin as a biomarker of kidney functional mass

Since it is exclusively produced in tubular cells, uromodulin may have a specific biomarker value for kidney function. The factors associated with uromodulin excretion were analyzed in the SKIPOGH and CoLaus cohorts [50]. In both studies, positive associations were found between uromodulin and urinary sodium, chloride, and potassium excretion and osmolality. In SKIPOGH, 24-h uromodulin excretion was positively associated with kidney length and volume and with creatinine excretion and urine volume. It was negatively associated with age and diabetes. Both spot uromodulin concentration and 24-h uromodulin excretion were linearly and positively associated (multivariate analyses) with eGFR < 90 ml/min per 1.73 m^2^. Age, creatinine excretion, diabetes, and urinary volume are independent clinical correlates of urinary uromodulin excretion. The associations of uromodulin excretion with markers of tubular functions and kidney dimensions suggest that it may reflect the distal tubular transport activity (e.g., reabsorption of NaCl and/or divalent cations) in the general population [50].

Further analyses in the SKIPOGH cohort evidenced that, in multiple linear regression analysis, 24-h urine uromodulin excretion was associated with the same predictors of nephron mass as identified by Denic et al. [9]. Age, female sex, and uric acid were negative predictors whereas height and birth weight were positive predictors of 24-h urine uromodulin excretion [45]. These data substantiate the measurement of urinary uromodulin levels as a useful surrogate marker for nephron mass. As such, higher uromodulin levels may indicate a higher functional reserve of the kidney with a lower risk of acute kidney injury (AKI): the more uromodulin you have, the higher functional reserve you have in case of post-operative AKI for instance [11, 53]. This situation should not be confused with the results of the GWAS studies indicating that the risk variants at the *UMOD* locus, which drive higher production of uromodulin reflected by higher levels in urine and blood, are consistently associated with an increased risk of CKD.

## Mendelian randomization to assess causality of uromodulin and CKD and hypertension

Following the GWAS and follow-up investigations pointing at *UMOD* variants driving higher uromodulin expression (and thus higher excretion in urine) as associated with an increased risk of CKD, the issue of causality was raised. In other words: does higher production of uromodulin drives a higher risk of kidney damage?

Since uromodulin is exclusively produced by kidney tubular cells, urinary uromodulin levels positively correlate with eGFR (< 90 ml/min/1.73 m^2^) and with kidney length and volume, whereas 24-h urinary uromodulin excretion is considered as a proxy of nephron mass [45, 50]. In line, higher urinary uromodulin levels, reflecting a higher functional reserve, are inversely correlated with the risk of kidney function decline in at-risk cohorts [20, 57]. The fact that lower urinary levels of uromodulin reflect decreased kidney functional mass is typical of reverse causation, i.e., when the disease affects the investigated risk factor. These elements constitute a bias when evaluating the causality of the urinary uromodulin levels, and thus of the *UMOD* variants, on the risk of CKD [13]. The facts that uromodulin also regulates blood pressure and that blood pressure and kidney function are interconnected further complicate the analysis [44, 47].

**Fig. 4:**
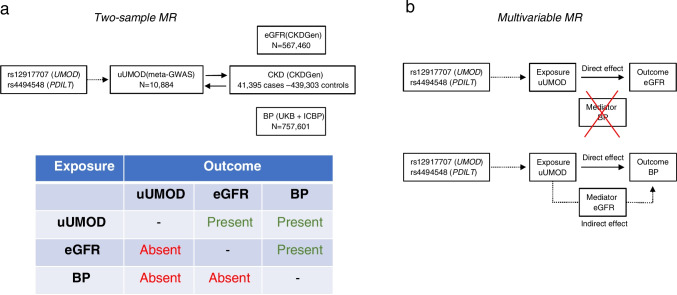
Mendelian randomization analyses supporting the association of higher levels of urinary uromodulin with lower kidney function and higher blood pressure. **a** Two-sample MR to assess the bidirectional causal effects between urinary uromodulin (uUMOD) and eGFR and between CKD and blood pressure (BP). The analyses were performed in the meta–GWAS for uUMOD involving 10,884 individuals; the CKD Genetics (CKDGen) consortium, a meta-analysis of 121 GWASs including 567,460 individuals of European ancestry; and a combined analysis of the UK Biobank (UKB) and the International Consortium of Blood Pressure (ICBP) GWAS, which amounted to 757,601 individuals. **b** Multivariable MR to assess direct and indirect effects of uUMOD on BP through eGFR and of uUMOD on eGFR through BP based on MR causal effects between the exposure, mediator, and outcome in the 2-sample MR analyses. The analysis suggests that the association of uUMOD with higher BP is partially through decreased kidney function, whereas BP does not appear to mediate the association of uUMOD with low kidney function. Modified from Ponte et al. [48] and Turner and Staplin [66]

The use of Mendelian randomization (MR) provides a way to assess whether the production of uromodulin, reflected by its urinary levels, is a true risk factor for CKD, and whether this potential association is related to blood pressure (Fig. 4). The MR method uses common genetic variants associated with the exposure (e.g., through GWAS), to test whether a given risk factor causes or aggravates a disease [55]. Recently, MR was used to clarify causality between urinary uromodulin levels, kidney function, and blood pressure in individuals of European descent [48]. The link between urinary uromodulin levels and eGFR was first investigated in CoLaus (*n* = 3851 available data). In observational data, higher urinary uromodulin associated with higher eGFR. Conversely, when using the *UMOD* rs12917707 as an instrumental variable in one-sample Mendelian randomization, higher uromodulin levels strongly associated with eGFR decline.

Interpretation of MR studies should integrate that genetic variants may have other effects beyond the studied biomarker—a situation called genetic pleiotropy. Since uromodulin modulates sodium transport and influences salt sensitivity [47], our MR analysis substantiated the genetic association between the main *UMOD* variant and blood pressure, in addition to CKD and eGFR [48]. We thus applied two-sample Mendelian randomization on four GWAS consortia to explore causal links between urinary uromodulin levels and eGFR, CKD risk (567,460 individuals) and blood pressure (757,461 individuals). Higher uromodulin levels significantly associated with lower eGFR, higher odds for eGFR decline or CKD, and higher systolic or diastolic blood pressure. Although the causal effects of urinary uromodulin levels on the risk of CKD are independent of blood pressure, the effect on blood pressure is mediated by eGFR [48]. These data support that genetically driven levels of uromodulin have a direct, causal, and adverse effect on kidney function outcome in the general population, not mediated by blood pressure (Fig. 4). They provide a strong rationale for investigating the mechanism by which elevated urinary uromodulin may contribute to CKD and whether a suitable modulator of uromodulin production/secretion may be identified [48, 66].

## The *UMOD* locus and adaptation against uropathogens

As discussed above, the *UMOD* variants associated with risk of hypertension and CKD in the general population increase the expression and urinary excretion of uromodulin [11, 13, 53]. Yet, population genetics investigations indicated that the T allele of the top *UMOD* GWAS variant, rs4293393, associated with CKD risk, is the ancestral allele and is kept at high frequency in most modern populations [22, 65]. In fact, the distribution of the *UMOD* ancestral allele does not follow the ancestral susceptibility model observed for variants associated with salt-sensitive hypertension, i.e., a higher prevalence of salt-retaining alleles in African compared to non-African populations, reflecting purifying selection outside of Africa [52]. Instead, the risk variant showed a significant correlation with pathogen diversity (bacteria, helminths) and prevalence of antibiotic-resistant UTIs. An inverse correlation between urinary levels of uromodulin and markers of UTIs was detected in CoLaus [22]. A prospective cohort study of elderly community-dwelling individuals found that those with urinary uromodulin concentrations in the highest quartile had a lower risk of UTI events than those in the lowest quartile, independent of classical UTI risk factors [19].

Uromodulin is known to show antimicrobial properties in urine, and *Umod*-knockout mice are more prone to UTIs induced by type‑1 fimbriated *E*. *coli* (FimH adhesin) than are their wild-type littermates [2, 35]. UTIs are among the most common bacterial infections, with high incidence and risk of recurrence in young women and potential complications that may reduce fitness. Pathogens and infectious diseases have imposed a strong selective pressure throughout human history [18]. Thus, the *UMOD* ancestral allele, driving higher urinary excretion of uromodulin, may have been kept at a high frequency because of its protective effect against UTIs [22]. This hypothesis is supported by structural and biochemical studies showing that the high mannose *N*-glycans at residue Asn275 in the cysteine-rich D8C domain of uromodulin are critical for FimH binding and thus prevention of the colonization of bladder epithelia by UPEC. The capacity of uromodulin to bind and aggregate bacteria is prevented by removal of the high mannose *N*-glycan or by competitive effect of an excess of d-mannose [68]. Of note, uromodulin was also able to aggregate other bacteria involved in human UTIs, including *Klebsiella pneumoniae*, *Pseudomonas aeruginosa*, and *Streptococcus mitis* [68]. These data demonstrated the role for uromodulin in defense against UTI through the capacity to aggregate uropathogens and to prevent their adhesion to the urothelium.

## Rare mutations in *UMOD* cause autosomal dominant tubulointerstitial kidney disease

In addition to common genetic variants associated with the risk of CKD and hypertension in GWAS, dominantly inherited mutations in *UMOD* are causing a rare form of kidney disorder leading to kidney failure.

Autosomal dominant tubulointerstitial kidney disease (ADTKD, MIM #16,200) is an increasingly recognized cause of end-stage kidney disease, characterized by tubular damage and interstitial fibrosis of the kidney in the absence of glomerular lesions. Affected individuals present with urinary concentrating defects, progressive chronic kidney disease (CKD), normal-to-mild proteinuria, and normal-sized kidneys, often with a positive family history [12, 14]. A relatively specific finding is hyperuricemia due to low fractional excretion of uric acid, causing gout, usually before the onset of CKD [41]. The disease invariably progresses to end-stage kidney disease (ESKD) in adulthood, with a penetrance of 100%. Rare (MAF < 10–4), dominant mutations in *UMOD* represent the most frequent cause of ADTKD (ADTKD-*UMOD*). More than 95% of the *UMOD* mutations associated with ADTKD are missense, often targeting cysteine residues and leading to the formation of uromodulin aggregates within the endoplasmic reticulum (gain-of-toxic function) with a sharp decrease of its excretion in urine. Over 100 mutations in *UMOD* have been associated with ADTKD-*UMOD*, with an overall prevalence of ~ 2% in patients with kidney failure, representing one of the most common monogenic kidney diseases [21, 24].

The pathogenic mechanism of ADTKD-*UMOD* is due to the toxic accumulation of mutant uromodulin in TAL cells, with ER expansion and parallel decreased in the urinary levels of the protein [12]. Studies of mouse models carrying uromodulin mutations confirm that intracellular accumulation of mutant uromodulin leads to ER stress, induction of the unfolded protein response, and subsequent tubular damage and interstitial fibrosis—substantiating the gain of toxic function mechanism in ADTKD-*UMOD* [53]. This mechanism suggests that decreasing the production of mutant protein, for instance by using antisense oligonucleotides, could be a strategy to slow the disease course. The kidney-specific expression of *UMOD*, and the mild phenotype in *Umod* knockout mice support the potential value of such an approach [12].

*UMOD* is thus implicated at both extremes of the genetic disease spectrum: ultrarare variants with large effect size (ADTKD-*UMOD*) and common GWAS variants associated with reduced eGFR and risk of CKD in the general population. Thus, disorders involving uromodulin production and/or excretion are of widespread relevance.

## Conclusions and perspectives

Thirty years after the identification of the first gene involved in an inherited kidney disease, the use of increasingly efficient and affordable genetic tools has allowed to increase diagnosis efficiency for rare kidney disorders, to clarify genetic heterogeneity and disease ontology, and to discover modifier genes involved in intrafamilial variability [10, 60, 67, 69]. The use of whole genome, SNP genotyping, and phenotype data has also helped to elucidate the role of complex genetic variation in the missing heritability observed for CKD and kidney-related traits. For instance, a recent study evidenced the influence of variable nucleotide tandem repeats (VNTRs) in *MUC1* with multiple kidney phenotypes [38].

Discovering new genes will drive multi-level studies substantiating cellular mechanisms and possible drug targets. These analyses should further decipher the continuum of genetic kidney disease risk, with genes involved from rare Mendelian disorders to common variation in the general population. Examples include genes involved in NaCl handling at the kidney tubule level (e.g., *SLC12A3*, *KCNJ1*, *SLC12A1*, *UMOD*), also relevant for blood pressure regulation in the population; genes involved in receptor-mediated endocytosis in the proximal tubule (e.g., *LRP2*, *CUBN*, *DAB2*), shown by GWAS to affect renal function and risk of CKD; genes involved in rare disorders of Ca^2+^ and Mg^2+^ handling (e.g., *CASR*, *TRPM6*, *CLDN14*, *CNNM2*), also associated with mineral homeostasis and metabolic traits in the general population [67]. The mechanisms sustaining the effect of rare and common variants in these genes or in additional genes identified by GWAS will provide insights into various aspects of kidney function.

Analysis of large genomic datasets suggests that genetic variants with intermediate effect sizes must bridge the gap between rare, high-effect variants causing Mendelian disorders and frequent, low-effect variants involved in complex diseases (Fig. 1). These intermediate-effect variants can lead to either non-fully penetrant Mendelian disease or to an oligo/polygenic model modifying disease expressivity [30]. Such intermediate-effect variants may also be part of the genetic continuum underlying CKD, based on the lack of rare, pathogenic variants in the majority of CKD patients [24] and the missing heritability in GWAS [70]. Recently, we identified and characterized intermediate-effect variants in *UMOD* contributing to CKD by crossing general population datasets with curated variants reported in ADTKD; analyzing biological and phenotypical effect sizes using in silico modeling, cell systems, and databases and biobanks; and validating the impact on kidney failure in the 100,000 Genomes Project and UK Biobank [43].

Obtaining genetic information in patients with rare kidney diseases is already substantiating precision medicine and will probably increase rapidly [10, 17, 36]. With larger GWAS and advanced statistical methods, polygenic risk scores (PRS) will develop, with the perspective of the early identification of subjects at risk of developing complex kidney diseases, before eGFR decline has manifested—offering the possibility of early intervention [27, 70, 72].
